# 

**Published:** 1894-05-26

**Authors:** 


CONTENTS.

**The General Medical Council** - - - - - - 155
**Researches on Hydrides and Hydrates.** By Sir Benjamin Ward Richardson, M.D., F.R.S. - - - - - - - - 157
**Practical Apects of Medical Science** - - - - - 158
**Modern Medico-Psychology and Psychiatry.** VI.—The Men Who have Made Modern Psychiatry - - - - - 159
**Studies in Applied Sociology.** IX.—"The Lives We Have Bid to Be." - - - - - - - - - - - 160
**Annotations.**—A Provident Dispensary Failure—Why is Suicide on the Increase?—The "Religio Medici" - - - - - 161
**Notes and News** - - - - - - - - - - 162
**The Book World of Medici**[illegible]**e and Science** - - - - 175
**Medical Vacancies and Appointments** - - - - - 176
**Medical Progress and Hospital Clinics—**
Pelvic Peritonitis and Its Treatment - - - - - - - 163
Exophthalmic Goitre and Its Relation to Diseases of the Thyroid Gland - - - - - - - - - - - - - 165
Medical Progress—Diseases of the Nervous System - - - 166
Progress in Surgery.—Spinal Surgery - - - - - - 168
New Drugs and Preparations - - - - - - - 169
New Appliances and Things Medical - - - - - - 170
**The Institutional Workshop—**
Hospital Administration—The Birmingham Hospital Saturday Fund - - - - - - - - - - - - - 171
Hospital Festivals and Meetings - - - - - - - 173
**Scraps and Gleanings** - - - - - - - - - 174

EXTRA SUPPLEMENT.—THE NURSING MIRROR.

**News from the Nursing World.**—Princess Mary at Southwark—Nurses at Windsor—A Council of Hospital Matrons—Nurses and Cooking—The Writers' Club—The Aid of Two Charities—A Sad Lesson—A New Home for Nurses—An Interesting Meeting—A Pioneer of Nursing—Royal British Nurses' Association—A Wise Example—Heroic Treatment—Short Items - - - - - - - - - - - - lxxi

**On General Nursing.** XIII.—Pneumonia (*continued*) - - lxxiii

**Women's Work.**—Work in the Civil Service - - - - lxxiv

**Novelties for Nurses,** lxxiv; **Notes and Queries** - - lxxviii

**Wants and Workers** - - - - - - - - - lxxiv

**A Visit t**[illegible] **Canada.** II.—The Sulphur Springs of Banff - - lxxv

**On Lecturing.** By One Who Has Done It - - - - - lxxvi

**The Proba**[illegible]**ione**[illegible]**'s Night Watch** - - - - - - - lxxvi

**Our Examinations** - - - - - - - - - lxxvi

**The Nur**[illegible]**ing of Army Sisters.** III. - - - - - - lxxvii

**Nursing in Ireland** - - - - - - - - - lxxvii

**For Reading to the Sick** - - - - - - - - lxxviii

**The Muse's Looking-Glass—Appointments** - - - - lxxix

**Everybody's Opinion—Where to Go** - - - - - - lxxx

